# Large Pelvic Cystic Mass of Mullerian Duct Origin with Involvement of Rectal Wall: A Case Report

**Published:** 2011-02-01

**Authors:** N Tayyebi Meibodi, R Mahdavi, M Montazer

**Affiliations:** 1 Department of Pathology, Mashhad University of Medical Sciences, Mashhad, Iran; 2 Department of Urology, Mashhad University of Medical Sciences, Mashhad, Iran

**Keywords:** Pelvic cyst, Mullerian duct, Immunohistochemistry, Progesterone receptors, Estrogen receptors

Dear Editor,

In the male fetus, Mullerian ducts degenerate leaving behind only remnants in connection with prostatic utricle and rete testis. Cysts of Mullerian origin are rare congenital lesions that are typically present as midline pelvic masses.[1][2] However, reports from scrotum, testis, epididymis, retroperitoneum and mediastinum also exist.[3][4][5][6][7] They usually occur in third and fourth decades of life with genitourinary problems being the most prevalent complaints.[1][2]

A 30-year-old Afghani construction worker man was admitted in urology ward of Imam-Reza Hospital, Mashhad, Iran in November 2008, with a fourmonth history of refractory constipation. No genitourinary problem was reported. The only positive finding in physical examination was a fluctuating perineal mass. Except for normocytic anemia and hematuria, all other tests were unremarkable.

**Fig. 1:**
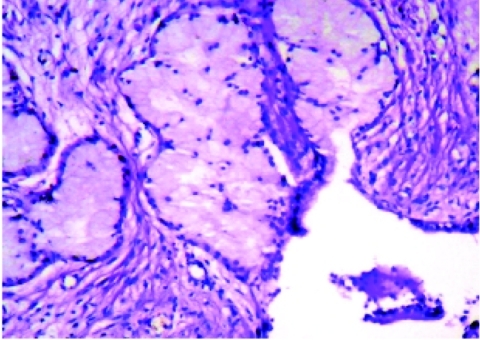
Cowper-like mucinous glands draining into the cyst (H and E, ×400)

Contrast-enhanced computed tomography (CT) scan demonstrated a midline multilocular cyst measuring 13 cm in greatest diameter located between urinary bladder and rectum with extension to prostate gland, perineum, cavernous bodies and scrotum. No obvious communication with prostate was seen. There was a diminished perirectal fat contrast indicating rectal adhesions. The aspirated fluid was spermfree, containing some scattered macrophages and epithelial cells. The culture of the fluid was negative. The patient underwent an operation that resulted in incomplete resection and partial colectomy due to extensive and severe adhesions to adjacent viscera and perineal skin. Microscopically, the cyst wall was lined with one layer of mucinous columnar epithelium. There was a proliferation of mucinous glands with complex branched ducts, similar to prebulbar (Cowper) glands, draining into the cyst (Figure 1). These glands were periodic acid-Schiff positive and resistant to diastase. Some projections of the cyst were inserted into the rectal muscular layer. Anaplastic changes or teratomic components were not found. An immunohistochemistry (IHC) profile was performed in order to differentiate the three main probable diagnoses of prostatic multilocular cystadenoma, benign multicystic mesothelioma and Mullerian duct remnant cysts. The specimen was completely nonreactive for either PSA or calretinin (excluding prostatic or mesothelial origin, respectively), but there were scattered PR-positive, and to a lesser extent ER-positive stromal cells (confirming the Mullerian origin). The epithelial lining of the cyst and the myoepithelial cells of the Cowper-like glands were reactive for cytokeratin and desmin, respectively. The patient did not report any further discomfort, afterwards.

The diagnosis of Mullerian duct cysts was classically made by considering the clinical, imaging and surgical investigations. However, recent studies highlights the importance of proper histological investigations especially the immunostaining for ER and PR.[8][9][10]
